# Effective radiation attenuation calibration for breast density: compression thickness influences and correction

**DOI:** 10.1186/1475-925X-9-73

**Published:** 2010-11-16

**Authors:** John J Heine, Ke Cao, Jerry A Thomas

**Affiliations:** 1 H. Lee Moffitt Cancer Center & Research Institute, Cancer Prevention & Control Division, 12902 Magnolia Drive, Tampa, FL, 33612, USA; 2 Via Christi Regional Medical Center, 929 N. St. Francis, Wichita, KS, 67214, USA

## Abstract

**Background:**

Calibrating mammograms to produce a standardized breast density measurement for breast cancer risk analysis requires an accurate spatial measure of the compressed breast thickness. Thickness inaccuracies due to the nominal system readout value and compression paddle orientation induce unacceptable errors in the calibration.

**Method:**

A thickness correction was developed and evaluated using a fully specified two-component surrogate breast model. A previously developed calibration approach based on effective radiation attenuation coefficient measurements was used in the analysis. Water and oil were used to construct phantoms to replicate the deformable properties of the breast. Phantoms consisting of measured proportions of water and oil were used to estimate calibration errors without correction, evaluate the thickness correction, and investigate the reproducibility of the various calibration representations under compression thickness variations.

**Results:**

The average thickness uncertainty due to compression paddle warp was characterized to within 0.5 mm. The relative calibration error was reduced to 7% from 48-68% with the correction. The normalized effective radiation attenuation coefficient (planar) representation was reproducible under intra-sample compression thickness variations compared with calibrated volume measures.

**Conclusion:**

Incorporating this thickness correction into the rigid breast tissue equivalent calibration method should improve the calibration accuracy of mammograms for risk assessments using the reproducible planar calibration measure.

## Background

Breast density is a significant breast cancer risk factor [1-3]. When estimating breast density from mammograms, the breast is considered as a two-component model consisting of adipose and fibroglandular (abbreviated as glandular hereafter) tissue to varying degrees. One method of measuring breast density uses binary labeling resulting in areas of radiographically dense tissue (glandular tissue) or adipose (non-dense) tissue. Breast density is then estimated as the ratio of the radiographically dense area to the total breast area (dense + adipose) [4-6]. Binary labeling techniques have repeatedly produced a measure that correlates well with breast cancer [2] without considering inter-image acquisition technique differences.

Recent work has focused on calibration to compensate for differences in the inter-image acquisition technique [7-15]. Calibration produces various standardized data representations by adjusting for variations in the target/filter combination, x-ray tube voltage, radiation exposure, and compressed breast thickness. By reducing measurement variation, calibration should produce a breast density measure that shows a stronger association with breast cancer in comparison with measurements derived without calibration. Moreover if the calibration measures prove viable, breast density assessments can be automated. Additionally, calibration applied at the local level supports the analysis of the calibrated measure's spatial distribution across the breast field of view that is not supported by the binary measure of breast density. In contrast, recent work [16,17] indicates that calibrated measures of breast density do not produce risk associations stronger than those produced without calibration. We hypothesize, calibration techniques will require further investigation and modification before they prove useful.

We have built our approach [8,9,18] upon earlier calibration work [10] in full field digital mammography (FFDM) to produce a normalized effective radiation attenuation coefficient representation for breast density. This work was developed under the assumption that known phantom heights corresponded with the mammography system compressed breast thickness digital readout value. Preliminary analyses showed that this assumption was not valid. Inaccurate compressed breast thickness represents an ongoing technical challenge in calibrated breast density research [19-21].

This paper addresses compressed breast thickness inaccuracies using deformable phantoms with the following objectives: (1) develop and evaluate a compressed breast thickness correction method that can be incorporated into the rigid breast tissue equivalent phantom calibration model, and (2) compare calibration representation reproducibility under compression thickness variations using known compositions. We used a surrogate breast model because the volumetric compositions were fully specified.

## Methods

To address the study objectives, a method was derived from the calibration methodology to characterize the compressed sample's spatial thickness variation. This method was evaluated under controlled conditions using modified (rigid) breast tissue equivalent phantoms with known height variations before addressing deformable samples. The rigid phantoms used for this work were purchased from Computerized Imaging References Systems (CIRS, Norfolk VA) and were described in our previous report [9]. These phantoms (non-modified) are standards in calibration research. We coupled this spatial thickness characterization with mechanical measurements of the compression paddle to construct a correction. The correction was evaluated within the calibration application using an alternative two-component deformable phantom model constructed with water and oil filled balloon phantoms that replicated patient imaging. We investigated the compression behavior similarity between mammograms and these deformable phantoms. This alternative model was then used to investigate the calibration representation reproducibility while varying the compression thickness.

### Imaging System

Imaging was performed with a General Electric Senographe 2000 D FFDM system, which is used for routine breast cancer screening examinations. The detector specifics were described previously [22]. All phantom images were acquired as left craniocaudal (LCC) views using a Molybdenum/Molybdenum target/filter combination and 26 kV x-ray tube voltage with 160 mAs, where mAs is the system readout value for the generated radiation. An extensive array of acquisition techniques was not required to validate and illustrate the main principles. The image data matrix is 1914 × 2294 pixels. A standard (x, y) positive coordinate system was used with the origin, (0, 0), located at the bottom left hand-corner of the displayed image, where × and y locations are integer valued pixel coordinates ranging from 0-1903 and 0-2293, respectively. The outside detector edge defined the y-axis (vertical direction). The following definitions were used below: x_max _= 1903, and y_max _= 2293. This system produces both raw and processed image data for display (for presentation) purposes. Raw image data, represented by r(x, y) below, was used for this work. This system is equipped with 15 × 20 cm^2 ^*rigid *compression paddle. The system compression force changes in 10 N increments with a minimum system readout value of 30 N (the first system readout value above 0.0). The system digital compression thickness readout value, defined as t_s _below, is cited in cm.

### Calibration Method

The calibration method described previously is outlined to support the subsequent analysis. The logarithmic response (LR) is given by LR(x, y) = ln[r(x, y)/mAs], where mAs is the system readout value and r(x, y) is the raw image. Calibration curves are generated by measuring the logarithmic response for both adipose and glandular tissue equivalent phantoms as a function of phantom height above the breast support surface using a specific reference mAs [8,9]. Two calibration points are required to standardize a given image. These points correspond to the logarithmic response for both the adipose and glandular tissue equivalent phantoms for the same image acquisition technique (same filter/target combination, kV and compressed breast thickness). These two calibration points (explained below) are generated with previously estimated calibration regression parameters

(1) $$\text{L}{\text{R}}_{\text{j}}(\text{x},\text{y},\text{t}=\text{T})=-{\text{μ}}_{\text{j}}(\text{x},\text{y})\text{T}(\text{x},\text{y})+{\text{l}}_{\text{j}}(\text{x},\text{y}),$$

where μ_j _is the effective radiation attenuation coefficient (cm^-1^) for either the glandular (μ_g _) or adipose (μ_f _) tissue equivalent phantoms, l_j _is the respective logarithmic intercept for either the glandular (l_g _), or adipose (l_f _) phantoms, and T(x, y) is the spatially dependent compressed breast thickness above the breast support surface (or deformed paddle height). We have demonstrated previously [8,9] that calibration curves measured over a wide range of phantoms heights [T in Eq. (1)] and modeled with the Eq. (1) form as function of T were well approximated as linear for a fixed x-ray tube voltage (kV) and target/filter combination (fixed beam condition). Likewise, the spatial dependencies for μ_j _and l_j _can also be dropped without introducing significant error. For each target/filter combination and kV setting, there is a unique set of four calibration parameters derived from regression analysis. These regression parameters (μ_j _and l_j _) are stored and used in the calibration application to generate the two required calibration points (discussed above). For a given kV and target/filter combination, a generalized (approximation) form of Beer's law holds by replacing the monochromatic radiation attenuation coefficient with the effective radiation coefficient as expressed in Eq. (1). The idealizations used to develop this model were also discussed previously [8-10] and are not addressed here. All calibration data was acquired with the reference exposure setting mAs = 160. For a given beam type, calibration data acquired with one reference mAs value is sufficient to generate calibration points for arbitrary mAs values. Because the calibration curves are linear, the calibration application takes a linear form for a given beam (given target/filter and kV). Dropping the spatial dependencies, the calibration mapping takes this form

(2) $$\text{P}\text{G}=\text{M}\times \text{L}{\text{R}}_{\text{a}}+\text{B},$$

where LR_a _is a measured arbitrary logarithmic response for the same compressed sample thickness, T, used in Eq. (1) and percent glandular (PG) defines the calibrated representation. To determine M and B, we use the adipose and glandular regression parameters with Eq. (1) to provide the two boundary conditions using the known percent glandular compositions (that is, when j = f, PG = 0, and when j = g, PG = 100), which gives

(3) $$\text{M}=100\times {\left[({\text{μ}}_{\text{f}}-{\text{μ}}_{\text{g}})\text{T}+({\text{l}}_{\text{g}}-{\text{l}}_{\text{f}})\right]}^{-1}$$

and

(4) $$\text{B}=\text{M}\times ({\text{μ}}_{\text{f}}\text{T}-{\text{l}}_{\text{f}}),$$

where LR_a _is a measured arbitrary logarithmic response for the same compressed sample thickness, T, used in Eq. (1). The choice to constrain the mapping between 0-100 was arbitrary. Thus, for a given beam-type, the mapping is a function of the four related spatially-static regression parameters and the variable T that has a spatial dependency in general. The mapping indicates that an arbitrary logarithmic response (a measured LR_a _) is a linear combination of the adipose and glandular response as defined in Eq. (1) for the same beam type. A variant of Eq. (2) is found by rescaling the percent glandular form, p = PG/100, which gives the effective attenuation coefficient representation

(5) $${\text{μ}}_{\text{e}}={\text{μ}}_{\text{g}}\text{p}+(1-\text{p}){\text{μ}}_{\text{f}}.$$

Equations (2) and (5) are planar representations. Spatial averaging [Eq. (2) or Eq. (5)] gives either the average percent glandular, <PG >, composition or equivalently the average effective radiation attenuation coefficient, < μ_e _>, for the sample, respectively.

### Inaccurate Compression Thickness

Inaccurate calibration of mammograms stems from applying Eqs. (1-4) to an arbitrary logarithmic response with the incorrect compression thickness. Thickness inaccuracies are due to both the compression paddle deformation/tilt and inaccurate (nominal) system compression thickness digital readout value. There is a rigid/non-rigid misalignment between the non-compressible tissue equivalent phantoms used to generate the height dependent calibration data, which have precise heights and plane surfaces, and the compressed breast thickness determined by the system value. We replicated this misalignment (described below in more detail) with deformable phantoms for the calibration data generation, estimating the compression thickness variation, and evaluating the thickness correction with calibration accuracy comparisons.

### Related Calibration Representations

Related calibration representations are developed for comparisons. The spatially distributed glandular height representation is given by

(6) $${\text{h}}_{\text{g}}(\text{x},\text{y})=\text{p}(\text{x},\text{y})\text{T}(\text{x},\text{y}),$$

where p was defined above. Using an alternative approach, some researchers estimate the glandular height as the calibrated measure [7]. Either the total glandular volume or average glandular-volume/pixel can be determined with Eq. (6). When the volume of interest projection contains n pixels with digital spatial resolution = d (the detector element spacing measured in length units), the average glandular-volume/pixel is given by

(7) $$<{\text{V}}_{\text{d}}>=\frac{1}{\text{n}}\sum_{\text{x},\text{y}}{\text{h}}_{\text{g}}(\text{x},\text{y}){\text{d}}^{2}.$$

Multiplying the above equation by n gives the total glandular volume. Other researchers use a normalized volume breast density measure [11] that we label as V_N_. V_N _is the total glandular volume normalized by the total volume considered. Using Eq.(6) and Eq.(7) with constant compression thickness gives

(8) $${\text{V}}_{\text{N}}=\sum_{\text{x},\text{y}}{\text{h}}_{\text{g}}(\text{x},\text{y}){\text{d}}^{2}/\text{n}\text{T}{\text{d}}^{2}=<\text{p}>.$$

The above expression is equivalent to <PG >/100, which has different dimensionality compared with Eq. (7). A similar [Eq. (8)] breast density representation [23] results using the total glandular height normalized by the total breast height (approximated by nT above). We compared these representations below.

### Thickness Variation Characterization

We derived a method to estimate the compression paddle deformation due to applied compression force using Eq. (1). This approach leverages the linear logarithmic response characteristic. Expressing Eq. (1) at some arbitrary × location, x_0 _, gives

(9) $$\text{L}{\text{R}}_{0}({\text{x}}_{0})=-{\text{μ}}_{\text{k}}\text{t}+{\text{l}}_{\text{k}}.$$

where the subscript defines previously estimated calibration parameters for an attenuating material referenced as k. We use k because the approach was evaluated first with the rigid glandular (modified) breast tissue equivalent phantoms (k = g) with known height variations before applying the technique to the deformable water phantoms (k = w) with unknown height variations. The logarithmic response at the adjacent value of × is similarly expressed

(10) $$\text{L}{\text{R}}_{1}({\text{x}}_{0}+\text{Δ}\text{x})=-{\text{μ}}_{\text{k}}(\text{t}+\text{Δ}{\text{t}}_{1})+{\text{l}}_{\text{k}},$$

where Δt_1 _is the height variation of the adjacent logarithmic response along the x-direction. The above expression can be extended to the next value, LR_2_(x+ 2Δx) evaluated at t+Δt_1_+Δt_2 _, and so on. The relative height variation n-pixels from x_0 _is then estimated by subtracting the zero-order term and dividing by the known effective radiation attenuation coefficient giving

(11) $${\text{H}}_{\text{y}}(\text{n})=\frac{\left(\text{L}{\text{R}}_{\text{n}}-\text{L}{\text{R}}_{0}\right)}{-{\text{μ}}_{\text{k}}}=\sum_{\text{i}=1}^{\text{n}}\text{Δ}{\text{t}}_{\text{i}}.$$

The logarithmic-intercept was eliminated because it is influenced by height uncertainty. Equation (11) gives the relative height variation from × = x_0 _to × = x_0 _+n pixels, orthogonal to the y-axis at a given y location. A one-dimensional profile is determined by letting n become an integer variable defined over a given x-range. The two-dimensional relative height surface

[H(x, y) ] was derived by applying Eq. (11) over an extended y-range. The H(x, y) surface describes the relative height (lower surface) of the compression paddle warp/deformation. We let x_0 _= 0, which keyed the analysis to x_0 _= 0 (detector/paddle front-edge). To reduce variation, operations along the × direction were performed using the average of a sliding 10 pixel window, maneuvered without overlap. The approach was applied along the y-direction by substituting y and Δy for × and Δx in the above development.

### Thickness Variation Characterization Evaluation Methods

**Figure 1:**
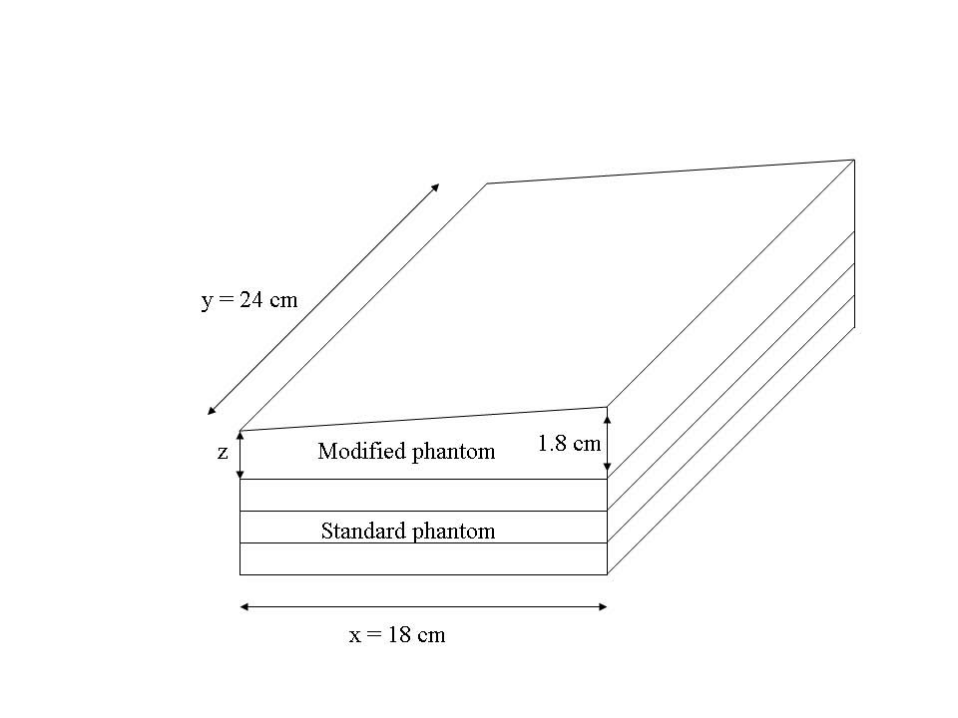
**Modified breast tissue equivalent phantoms**. This illustration shows a modified phantom (top) stacked upon three standard phantoms. These modifications give a constant height gradient = z/1800 (mm/pixel) in the x-direction for z ranging from 1-4 mm (in 1 mm increments).

The H(x, y) method (described above) was applied to modified glandular breast tissue equivalent phantoms for evaluation purposes before applying it to unknown thickness variations. Standard 2 cm (rigid glandular breast tissue equivalent) phantoms were machined by the phantom manufacturer (CIRS) along one face forming slanted-plane phantoms (slant-phantoms) with constant height gradients rising from 1.7-1.8 cm, 1.6-1.8 cm, 1.5-1.8 cm, and 1.4-1.8 cm along the x-direction (inclined planes rising from 1 mm to 4 mm in height along the x-direction). The modification is shown in Figure 1. Modified phantoms were used in combination with the standard (uniform thickness) glandular tissue equivalent phantoms to build slant-phantoms of varying total heights as specified in Table 1 (see Figure 1). The lower side of the slant-phantom was aligned along × = 0 to simulate the upward paddle bulge caused by compression near the central region of the paddle. For evaluation, H(x, y) was compared with the known height variation of the slant-phantoms. The effective radiation attenuation coefficient for the glandular equivalent phantom, which was measured previously using regression analysis for 1-6 cm phantom heights [9] for the respective acquisition technique (μ_g _= 0.833 cm^-1 ^), was used with Eq. (11).

**Table 1:** Modified breast tissue equivalent phantom characterization

Standard phantom		1 mm slant		2 mm slant		3 mm slant		4 mm slant	
**mean** **(SD)**	**range** **(cm)**	**mean** **(SD)**	**range** **(cm)**	**mean** **(SD)**	**range** **(cm)**	**mean** **(SD)**	**range** **(cm)**	**mean** **(SD)**	**range** **(cm)**

0.012 (0.005)	1	-0.005 (0.008)	1.7-1.8	-0.004 (0.010)	1.6-1.8	0.005 (0.009)	1.5-1.8	-0.008 (0.008)	1.4-1.8
0.009 (0.006)	2	-0.009 (0.008)	2.7-2.8	-0.010 (0.008)	2.6-2.8	-0.011 (0.009)	2.1-2.4	-0.022 (0.006)	2.4-2.8
0.003 (0.006)	3	-0.010 (0.008)	3.2-3.3	-0.015 (0.008)	3.1-3.3	-0.021 (0.009)	3.1-3.4	-0.027 (0.007)	3.0-3.4
-0.016 (0.006)	4	-0.018 (0.008)	3.7-3.8	-0.022 (0.008)	3.6-3.8	-0.035 (0.011)	3.5-3.8	-0.038 (0.009)	3.5-3.9
-0.023 (0.009)	5	-0.025 (0.009)	4.7-4.8	-0.029 (0.009)	4.6-4.8	-0.046 (0.013)	4.5-4.8	-0.049 (0.013)	4.4-4.8
-0.032 (0.012)	6	-0.028 (0.010)	5.3-5.4	-0.034 (0.011)	5.2-5.4	-0.051 (0.015)	5.3-5.6	-0.0632 (0.018)	5.4-5.8

The difference image, d(x, y), distribution quantities for the four slanted-plane glandular equivalent phantoms and for the standard (zero-slant) phantom are provided. The 1-4 mm slant entries (top row) refer to z in Figure 1. The mean value and standard deviation (SD) for the d(x, y) difference metric [see Eq. (12)] pixel distributions are provided for each total phantom height and type expressed in cm. The range column gives the total phantom height change measured from the breast support surface to the front-edge and back-edge of each phantom configuration, respectively.

### Non-rigid Breast Simulating Phantoms

Phantoms were constructed to approximate the shape and deformable behavior of breast tissue. Breast tissue is assumed deformable but non-compressible [24]. We use *compress *herein to imply the compression paddle operated and the phantom (or breast) deformed accordingly. Thick walled balloons were filled with either distilled water (water), vegetable oil (oil), or water/oil mixtures with known proportions to simulate breast compression.

The breast (CC orientation) is a deformable organ that is relatively pliable. Therefore, the paddle plane was treated as a plate that warps when loaded. The applied compression force is a measure of the sample's resistance (load) to deformation distributed over the contact area transmitted through the strained paddle to the compressor arm. It is the rigidity/elasticity of the paddle plane in combination with that elastic resistive force offered by the compressed sample that determines the final paddle warp (bulge) required for static equilibrium during imaging. Although the breast is a complicated mixture of tissue with varying elastic properties, as an approximation we assume the entire organ behaves as composite deformable body with its own global elastic property. This is supported by earlier work showing that breast compression and mammographic density are unrelated [25]. To show that the deformable phantoms reasonably approximate the resistance offered by the breast undergoing compression, two requirements should hold: (1) the applied force and paddle contact area should be approximately coincident, and (2) the contact area geometry should be similar. Thus, the actual compression thickness similarity is irrelevant for this comparison. If we assume the paddle surface lies in a plane with a given surface area when not stressed, the corresponding warped (stressed) surface will have a slightly greater surface area than that calculated with its x-y dimensions due to the curvature induced by load. In the area calculations, we used the x-y planar dimensions of the paddle, which neglects the increased surface area.

**Figure 2:**
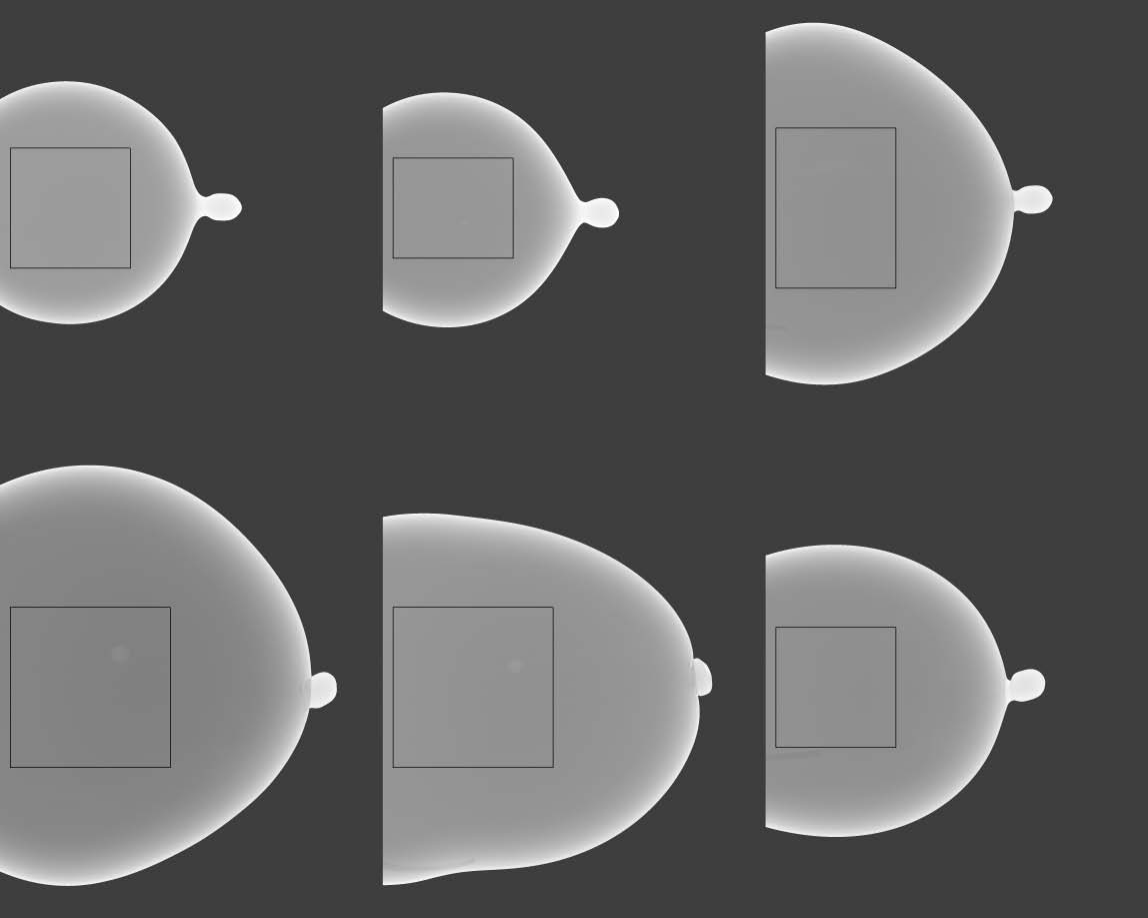
**Set of six water filled phantoms**. These phantoms were used to assess the compressed sample thickness variation as a function of compression force range. Thirty-five images were acquired from these six phantoms over a range of compression forces. The system readout thicknesses for these displayed examples were between 3-4 cm.

To estimate the breast-paddle contact area, 110 FFDM CC view study mammograms were selected consecutively from the database. The CC view has reduced chest muscle interference and is therefore the preferable view [26]. This is not a limitation, because we are only considering CC views in this calibration work for the same reason. The breast area was automatically segmented from the background using a simple pixel threshold method based on the acquisition technique. We estimated that eroding the breast outline by 21 percent along a radial direction located the breast-paddle contact area, which is an average approximation. This estimate comes from prior user-assisted analysis of estimating the paddle-contact area by evaluating line-profiles through the breast (100 mammograms) to determine the location of the intensity drop-off due to the breast curvature. The radial direction origin was centered at × = 0, and y = vertical direction centroid of the segmented breast calculated with pixel values = 1 (on the segmented breast area). The contact areas for the deformable phantoms shown in Figure 2 were estimated with a threshold approach based on their characteristic *single valued *pixel value distribution over the paddle contact area. We compared the compression force and contact areas of the mammograms with the deformable phantoms to assess similarities.

### Compression Paddle Measurements

**Figure 3:**
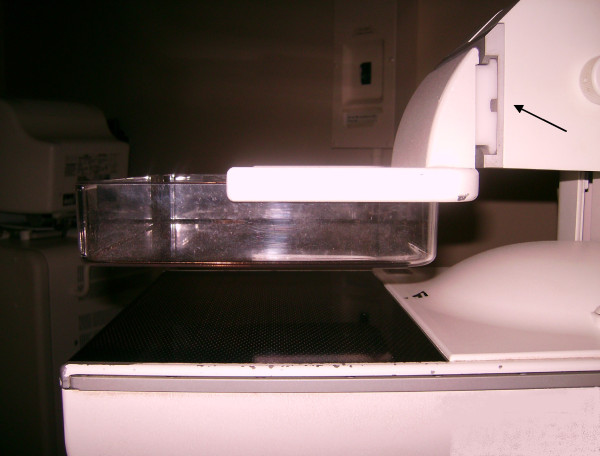
**Compression paddle**. The paddle is in the relaxed mode. Play in the connection (arrow) allows the paddle to tilt upward (front of the detector) when there is upward resistance to the downward compression force. The sidewalls add to the paddle perimeter rigidity.

The compression paddle's orientation to the breast support surface and system thickness readout were characterized under conditions that simulated patient imaging using (compressing) water filled phantoms. The paddle was inspected manually to assess its pliability. The resting paddle is shown in Figure 3. Measurements from the breast support surface to the paddle *corners *were taken under various conditions and compression forces by compressing the water-filled phantoms. These measures were taken repeatedly over an 18-month period. As an approximation, we assumed the detector and paddle outlines were aligned (the paddle area dimension is less than the breast support surface dimension). A feeler gauge technique, similar to that used to gap spark plugs, was used to make these compression paddle height measurements. Combinations of materials with precise thicknesses ranging in thickness from 1 mm to 10 mm were used to measure the distance between the breast support surface and paddle at each corner. Perimeter bulge was assessed with a straightedge along the x-direction perimeter (for y = 0 and y = y_max_) from the bottom side of the paddle. The paddle front-edge perimeter flex (along y for × = 0) was difficult to assess properly with mechanical measurements due to both the positioning of the deformable phantoms and the construction of the paddle near the edge from within the top side. Therefore, Eq. (11) was modified (along y) and used to better estimate the degree of flex along the paddle front-edge perimeter. Secondly, the compression paddle bulge was coarsely characterized using a similar feeler gauge technique applied within the central portion of the paddle (top side) along the y-direction. These mechanical measurements in combination with the H(x, y) analysis were used to construct a thickness correction surface.

### Paddle Deformation Characterization

A set of six water filled phantoms was imaged over a range of compression forces resulting in 35 images. These were used to characterize the compression thickness spatial variation due to the compression paddle plane deformation (bending or warping) by applying the H(x, y) analysis. In this analysis, we used the estimated regression parameters for water (k = w) to estimate the H(x, y) surface [see Eqs. (9-11)] because the phantom conforms to the warped compression paddle surface (the compression thickness surface). These phantoms were filled with arbitrary volumes of water ranging from 500-1200 ml to simulate various breast sizes as shown in Figure 2. The analysis was constrained to the outlined regions to avoid the curvature regions. These regions were 500 ×500 pixels or larger. Phantoms were imaged over a range of compression forces (summarized below). Phantoms were placed on the breast support surface in the central portion of the detector in the y-direction by observation to simulate patient positioning. The effective radiation attenuation coefficient for water was estimated with methods described below using the Eq. (1) form as the regression model. The H(x, y) method was used to locate the maximum bulge heights and positions. Regression analysis was used to determine the relationships between the compression force and the paddle bending characteristics.

### Thickness Correction

The thickness correction was developed using the two forms of measurements outlined above after validating the H(x, y) approach. The paddle bending summaries (from above) were joined with the paddle perimeter measurements. These measures, in combination, were used as boundary conditions to construct the polynomial thickness correction as a function of compression force.

### Alternative Calibration Model and Correction Evaluation Methods

**Figure 4:**
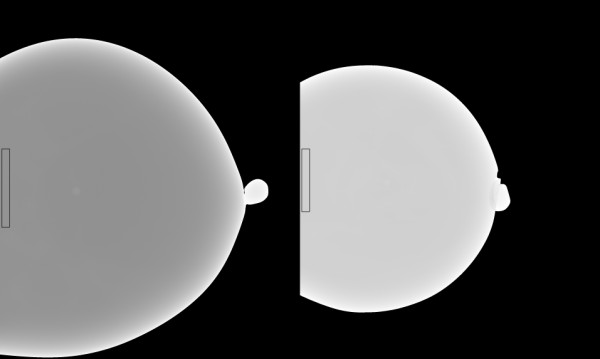
**Reference phantoms**. The water (left) and oil (right) phantoms were used as calibration references. Calibration parameters were generated from the outlined strips near the detector edge of 50 ×500 pixels (water) and 50 × 400 pixels (oil) to eliminate compression thickness uncertainty.

We used deformable phantoms to construct an alternative two-component model to (1) simulate calibrating mammograms, (2) duplicate the rigid/non-rigid misalignment, and (3) evaluate the thickness correction. Two additional calibration references phantoms were constructed with either water or oil with arbitrary volumes. Calibration curves were generated for each reference phantom shown in Figure 4 as a function of compressed phantom thicknesses (2.5-6.5 cm range). The outlined regions (small strips) were used because the compressed phantom thickness was known for these regions without applying the correction using t_s _+0.5 cm for the entire strip thickness (demonstrated below). Restricting the analysis to this strip simulated the methods used for generating the breast tissue equivalent regression parameters (using rigid phantoms with flat surfaces and precise heights). The water and oil regression parameters were used in Eqs. (1-4) as alternatives for the tissue equivalent parameters. These alternative parameters were used to initialize M and B as described above. Two additional mixture phantoms (deformable) were constructed with measured (known) volumetric proportions of water and oil to assess the thickness correction within the calibration application. These mixture phantoms were 34% and 31% water by volume (34/66 and 31/69 water/oil mixtures). The 34/66 mixture contained 300 ml water and 590 ml oil, whereas the 31/69 mixture contained 200 ml water and 441.5 ml oil. We estimated a 2% error in the water percentage for either mixture. The mixture phantoms were calibrated with and without the correction for comparison. Because these calibration parameters were generated with minimal height uncertainty within the strips (known uniform heights determined by the mechanical measurements), they were used to approximate the rigid/non-rigid misalignment. Calibrating the mixture balloon phantoms with the system readout height captures the rigid/non-rigid misalignment that occurs when calibrating mammograms using the system breast thickness readout height (nominal height); this quantity (incorrect height) is then used to generate the two required calibration points (incorrect points) with the phantom (with precise uniform heights) regression parameters. Although water and oil were used to develop an alternative two-component system, the mapping in Eq. (2) was referred to as percent glandular below rather than the percent water mapping because these two mappings are isomorphic.

### Standardized Representations Analysis

We used the alternative two-component system to investigate the various calibration representations and determine their reproducibility under varying compression forces. The 34/66 mixture was calibrated for three system compression readout thicknesses: 5.0 cm, 4.4 cm, and 3.8 cm. This simulated imaging the same patient at different times with varying compression forces. The total glandular volume, average glandular-volume/pixel, and percent glandular representations were calculated using Eqs. (2-8) and compared. The similarity between the effective x-ray attenuation coefficient representation expressed in Eq. (5) and the percent glandular representation expressed in Eq. (2) was demonstrated with regression analysis by calibrating the 34/66 mixture over a range of compression thicknesses. Both the percent glandular and glandular descriptions are used below for consistency with the understanding that they apply to water content for this work only.

## Results

### Thickness Variation Characterization Validation

The H(x, y) method was evaluated with modified breast tissue equivalent phantom imaging (Table 1). This analysis was constrained to large rectangular regions of 1000 × 1600 pixels centered on the detector in the y-direction aligned with the front edge of the breast support surface. The difference image

(12) $$\text{d}(\text{x},\text{y})=\text{H}(\text{x},\text{y})-{\text{H}}_{\text{T}}(\text{x},\text{y}),$$

was used for comparison, where H_T_(x, y) is the respective theoretical relative surface generated with the known constant height gradient for a given slant-phantom. The d(x, y) pixel distributions were summarized for all examples in Table 1. The average deviation of d(x, y) was generally less than 0.5 mm and not dependent upon the total phantom height.

### Compression Paddle Assessment

By physical inspection, the paddle plane has a stiff-membrane characteristic that permits constrained flexing. As shown in Figure 3, the sidewalls provide rigidity to the perimeter. Exerting spatially limited pressures at arbitrary locations within the plane induces similar bulge profiles with crests about the midlines. The *plane *of the paddle also has an upward curvature (about 1 mm crown) when resting with the maximum at approximately 73-75 mm from the chest wall slightly below the y-midpoint. We made mechanical measurements of the compression paddle perimeter repeatedly over an 18-month period. These measurements were consistent in both distance from the breast support surface and compression force. Surface flexing had negligible effects about the paddle perimeter in the x-direction at y = 0 and y = y_max _due to the paddle sidewalls (Figure 3). The paddle perimeter tilts in the x-direction when experiencing compression resistance. The relative perimeter elevation (measured in cm) was approximated by this expression

(13) $${\text{t}}_{\text{x}}=-0.0001568\text{x}+0.5.$$

**Figure 5:**
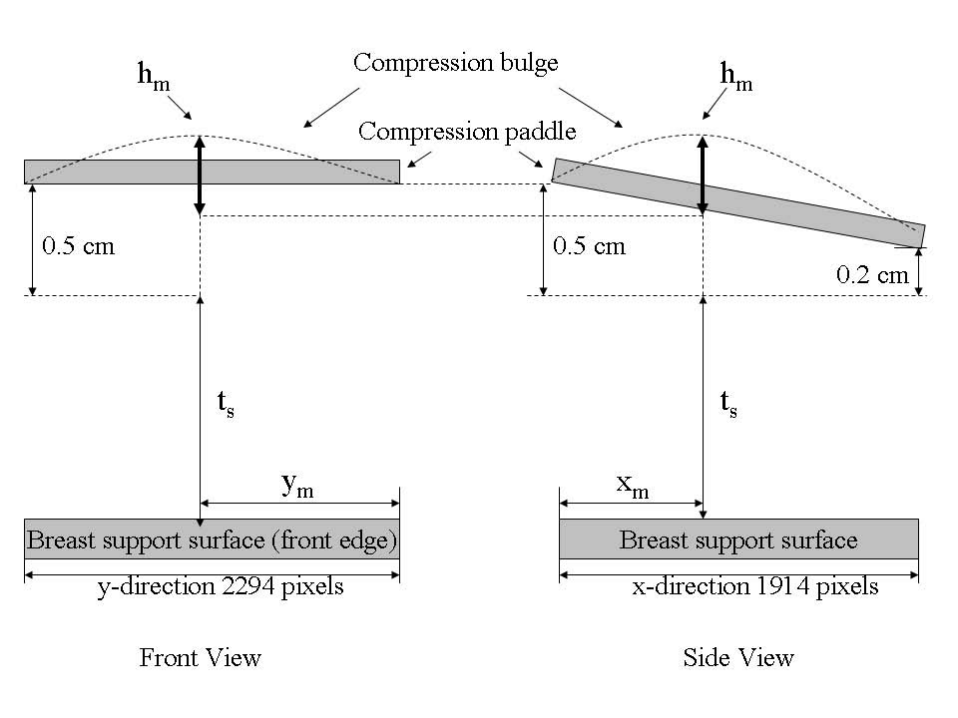
**Compression paddle perimeter-breast support surface illustration**. Various distances for the paddle perimeter assessments are illustrated with an arbitrary bulge. Adding 0.5 cm to the system compression thickness readout value, t_s _, gives the corrected height along the front edge of the breast support surface (left figure). The right figure shows the paddle tilt along the x-direction relative to t_s_. The paddle maximum bulge height (h_m_), located at (x_m_, y_m_) was estimated relative to paddle-perimeter height at × = x_m _for each of the phantom images that are summarized in Table 2.

The absolute perimeter height (cm) was given by t_x _+ t_s_. Equation (13) shows an upward paddle tilt toward the front edge of the detector, which is not present without applied compression force. We approximate less than ± 1.0 mm uncertainty in all measurements due to both measuring error/resolution and torque exerted on the paddle due to the position of the phantom. The arrow in Figure 3 points to the paddle triangular slide connection. The upward deflection is due primarily to the slack in this connection. Play in the slide allows the entire paddle plane to deflect upward in accord with the above relation occurring at less than 3 dN before bending occurs. Therefore, we assumed the paddle tilt was a maximum when imaging. The coarse measurements taken inside the compression paddle with the straightedge technique indicated the paddle surface bulge ranged from (0-4) mm from small to large compression forces up to 15 dN. These coarse measurements showed there is one maximum paddle bulge height that is a function of compression force. Figure 5 shows the relevant elevations of the paddle perimeter with respect to the breast support with an arbitrary bulge profile. We used Eq. (13) with the maximum bulge height and position coordinates as boundary conditions for the thickness correction.

**Figure 6:**
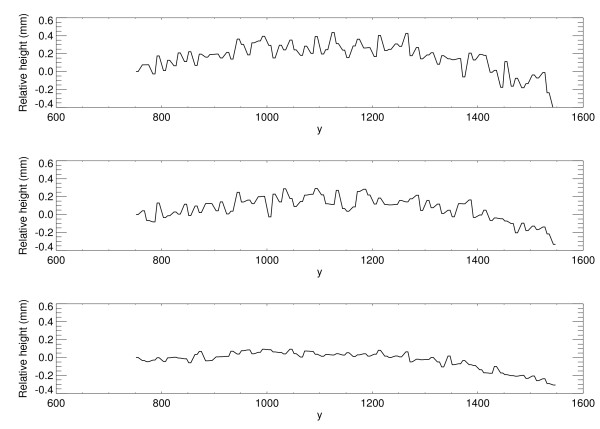
**Compression paddle deformed front-edge profiles**. Three one-dimension compression paddle profiles along y-direction at × = 20 are shown, which were estimated by modifying Eq. (11) to analyze bulge along the y-direction near the paddle front perimeter. The paddle perimeter flex is less than 0.4 mm. The compression forces (dN) were 4.0, 6.0, and 8.0 from top to bottom, respectively.

The modified form of Eq. (11) was used (along y) to assess the degree of bulge along the paddle front-edge perimeter. Figure 6 shows three one-dimensional profiles along the y-direction (x = 20) for a typical deformable phantom for three compression force levels. The curvature was less than 0.4 mm over an 8 cm span, which was negligible. Similar findings resulted when applying the analysis to the other water phantoms (from the 35 images). Therefore, we used Eq. (13) as an approximation for all y at × = 0 and × = x_max_. The maximum upward paddle height due to tilt alone was estimated with Eq. (13) for × = 0, which gives 0.5 cm (above t_s_).

### Paddle Deformation Characterization

**Figure 7:**
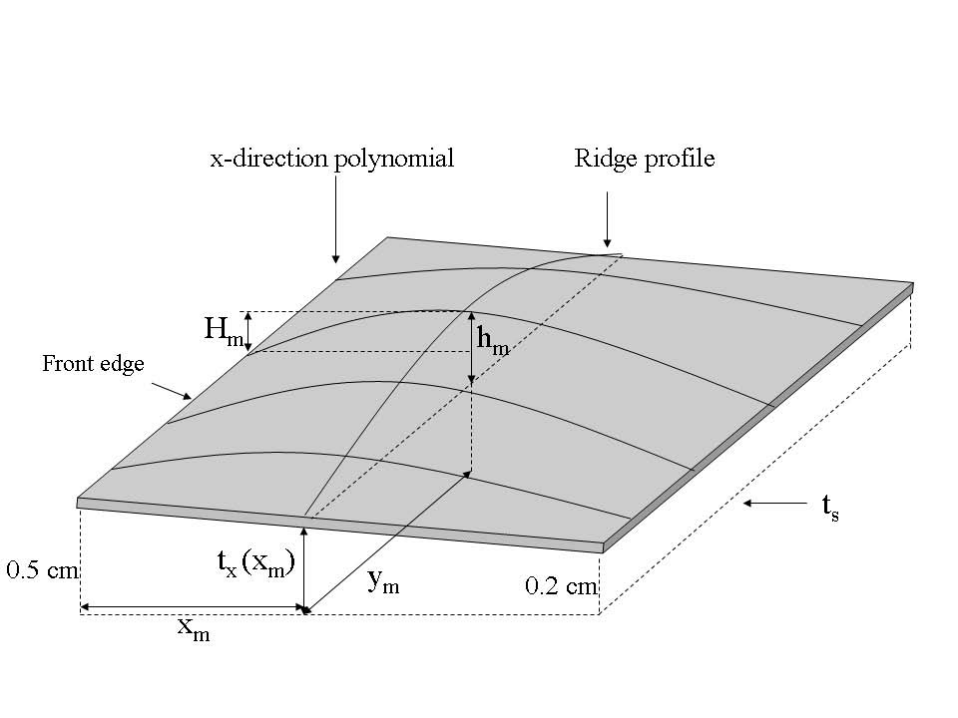
**The correction model**. All relevant measured distances, positions, ridge-profile, and separable x-direction polynomials are labeled on this correction surface illustration. The dashed line represents system thickness readout plane with t_s _(system readout height) parallel to the breast support surface. The ridge-profile runs along the y-direction at × = x_m _with a maximum height h_m _located at (x_m_, y_m_) measured above the perimeter height at x_m_. H_m _is the height above t_s _+0.5 cm measured from H(x, y) that was used to derive h_m_. A given x-direction polynomial was constructed with the position and height of the ridge-profile at the intersection of the two polynomials along with the relative paddle parameter heights at × = 0 and × = x_max_, which are 0.5 cm and 0.2 cm, respectively.

**Figure 8:**
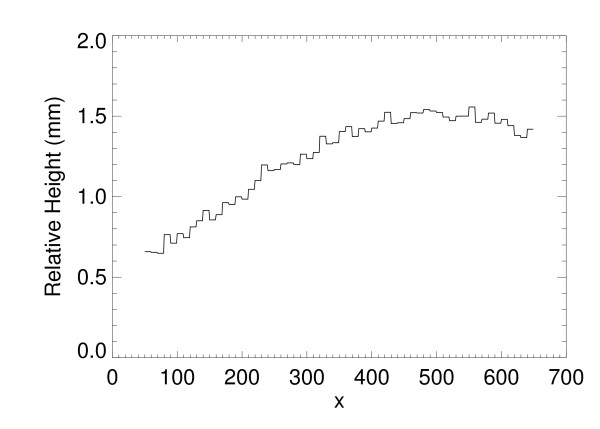
**Surface Profile**. This shows one dimensional H(x, y) profile through the maximum bulge height, H_m _(as well as h_m_) along the x-direction. The system thickness readout value was 3.2 cm with 7 dN compression force.

**Figure 9:**
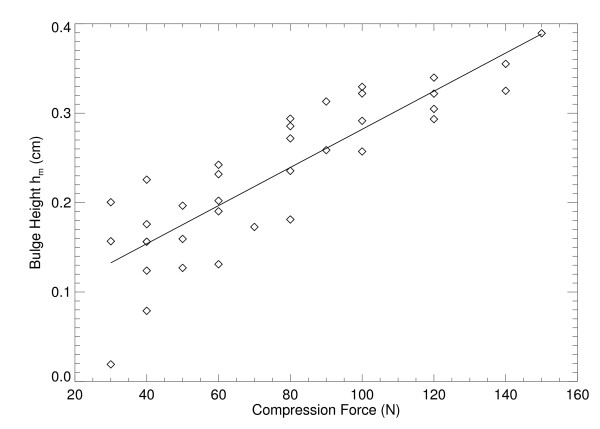
**Bulge height regression**. This shows the fitted (solid) linear relationship between the maximum bulge height (diamonds) as a function of compression force for the 35 water filled phantom images.

**Figure 10:**
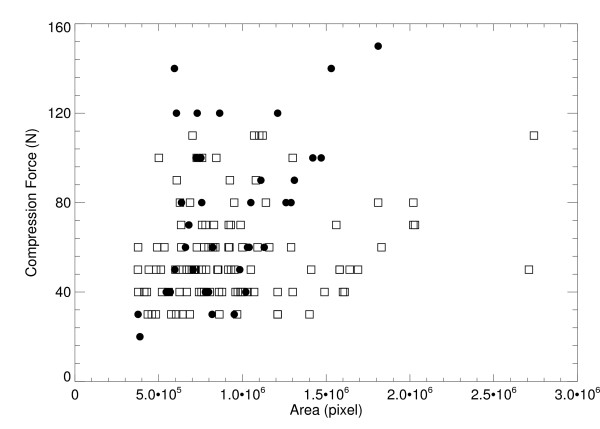
**Compression force contact area comparison**. This shows the force and contact area comparison for the study mammograms (squares) compared with the deformable water phantoms (filled circles) summarized in Table 3.

We applied the H(x, y) analysis to the collection of 35 water phantom images. For each H(x, y) surface, the coordinates, (x_m _, y_m_), and the maximum bulge height, h_m_, were estimated as a function of compression force (F_n _= system compression force readout quantity). The relevant parameters and distances are shown in Figure 5 and in Figure 7. The estimated maximum bulge height (h_m _) quantities are relative to Eq. (13) evaluated at × = x_m_. The H_m _distance is the maximum bulge height estimated with H(x, y). This is the distance above the front-edge of the compression paddle (above t_s _+ 0.5 cm) as illustrated in Figure 7. Figure 8 shows a one dimensional profile through H(x, y) along the × direction that intersects h_m _for the second phantom shown in Figure 2. The h_m _- F_n _regression plot is shown in Figure 9, and the related regression analysis is summarized in Table 2, which shows h_m _is well approximated as a linear function of F_n. _The R-square value indicates the model validity. Figure 10 shows the F_n _- A_n _scatter plot that compares the 110 mammograms (squares) and the 35 deformable phantom (filled circles) images. Summaries of the water phantom characteristics are listed in Table 3. For reference, the average maximum distance from the × = 0 to the eroded breast border for 110 mammograms was approximately 81 mm with a 24 mm distribution standard deviation, whereas the estimated quantities for the balloon phantoms were 86 mm and 60 mm, respectively (Table 3). For this system, the average compression force was estimated with 395 mammograms: <F_n_> = 57 N (distribution standard deviation ≈ 21 N). Eighty-eight percent of these images were within the 3-8 dN range. Because (1) the mammogram samples encapsulate the deformable phantom samples in Figure 10 over the 3-9 dN range, and (2) both the mammogram and phantom borders are approximately semi-circular, we conclude the two requirements specified earlier were approximately met, and their compression properties are similar. In summary, for the correction (from Table 3), we used y_m _= 2294/2 (approximation) and the average value of x_m _for bulge height positions independent of F_n_. The value of h_m _was generated for each specific F_n _in the correction construction using the parameters in Table 2.

**Table 2:** Bulge height and compression force regression analysis

Independent variable	Dependent variable	R-square	Intercept	Slope
compression force	bulge height (h_m_)	0.84	0.069 cm	0.002 cm/N

This gives the regression summary for the 35 water filled phantom images. It shows the bulge height (h_m_) is approximately a linear function of compression force.

**Table 3:** Water filled phantom characteristics

	Comp. Force (N)	x_m_ (pixels)	y_m_ (pixels)	h_m_ (mm)	H_m_ (mm)	max x-distance (mm)
mean	77.4	568.0	1169.1	2.37	1.48	86
SD	34.1	109.9	111.2	0.78	0.66	60

This gives the distribution means and standard deviations (SDs) for the compression force, maximum bulge height coordinates (x_m_, y_m _)_, _the bulge height (h_m_), the related H_m _height, and the maximum distance along the x-direction phantom/paddle contact (last column).

### Thickness Correction Construction

The compression paddle measurements provided four boundary conditions for constructing the thickness surface correction. Therefore, we used separable third degree polynomials for the correction. First, a one dimensional ridge-polynomial (Figure 8) along the y-direction was constructed that passes through the maximum bulge height and coordinates

(14) $$\text{R}({\text{x}}_{\text{m}},\text{y})={\text{c}}_{0}+{\text{c}}_{1}\text{y}+{\text{c}}_{2}{\text{y}}^{2}+{\text{c}}_{3}{\text{y}}^{3}.$$

The bulge coordinates, (x_m _, y_m,_), and associated height, h_m _, provided two interior boundary conditions. There is one ridge-profile per correction surface. Two other boundary conditions resulted from matching the ridge-profile height with the measured compression paddle perimeter height at (x_m_, 0) and (x_m_, y_max_). These four boundary conditions defined the coefficients in Eq. (14): (1) R(x_m_, 0) = t_x_(x_m_), (2) R(x_m_, y_max_) = t_x_(x_m_), (3) R(x_m_, y_m _) = h_m _+ t_x_(x_m_), and (4) dR/dy = 0 at (x_m_, y_m_). The two-dimensional correction surface was generated as a series of one-dimensional cubic polynomials in the x-direction using the ridge-profile intersection as the maximum height and position boundary conditions as shown in Figure 7 (interior boundary conditions). Two other boundary conditions were found by matching the x-direction polynomial height with the measured paddle parameter height at × = 0 and × = x_max_. The two-dimension relative correction surface was generated by constructing a one-dimensional x-polynomial for each value of y expressed as

(15) $${\text{t}}_{\text{c}}(\text{x},\text{y})={\text{a}}_{0}+{\text{a}}_{1\text{y}}\text{x}+{\text{a}}_{2\text{y}}{\text{x}}^{2}+{\text{a}}_{3\text{y}}{\text{x}}^{3},$$

where the coefficient subscripts include the y dependency for given profile. For fixed y, the coefficients were defined with these boundary conditions: (1) t_c_(0, y) = t_x_(0), (2) t_c_(x_max, _y) = t_x_(x_max_), (3) t_c_(x_m_, y) = R(x_m_, y), and (4) ∂t_c_/∂x = 0 at × = x_m_. A 20 pixel constant (relative) height margin (x = 0-19) was set equal with t_x_(0) to approximate the rigidity of the paddle front-edge. This was neglected at the other three perimeter segments due to the large distances from the bulge height position. The corrected compressed sample thickness in cm was expressed as t(x, y) = t_s _+ t_c_(x_, _y).

### Thickness Correction Evaluation

We evaluated the compressed thickness correction within the calibration application.

Regression (calibration) parameters were measured from the regions (strips) outlined in the water/oil reference images shown in Figure 4 over a range of compressed sample thicknesses. These regions were divided into 50 × 50 pixel sub-regions. The average pixel value for each sub-region was used to generate the logarithmic response for each region and phantom thickness. Phantom thicknesses were derived from the mechanical measurements given by t = t_s_+ 0.5 cm, which is a close approximation because t_x_(50) ≈ 0.50 cm. Linear regression was applied to each sub-region. The summarized regression distribution quantities are provided in Table 4 with t_s _for reference. Spatial averages of the regression parameters were used in Eqs. (3-4) with Eq. (2) to calibrate the mixture examples.

**Table 4:** Calibration regression parameters

Reference Phantoms	< μ > (cm^-1^)	< Log-Intercepts > (l)
Water_s_	0.708 (0.001)	4.056 (0.09)
Water_r_	0.708(0.005)	4.090 (0.02)
Water_nc_	0.684 (0.014)	3.884(0.09)
Oil_s_	0.459 (0.001)	4.957(0.03)
Oil_r_	0.455 (0.002)	4.95(0.02)
Oil_nc_	0.447 (0.001)	4.669(0.03)

The calibration regression parameter distribution means <μ> and standard deviations (parenthetical entries) for the pure water and oil deformable phantoms are tabulated. The subscripts (s, r, nc) define (1) quantities generated from the narrow strips (s) shown in Figure 4, (2) quantities generated from the larger region (r) of interest (25 cm^2^) using the compression thickness correction, and (3) quantities generated from the larger region of interest without applying the correction (nc) using the system thickness readout values, respectively.

**Figure 11:**
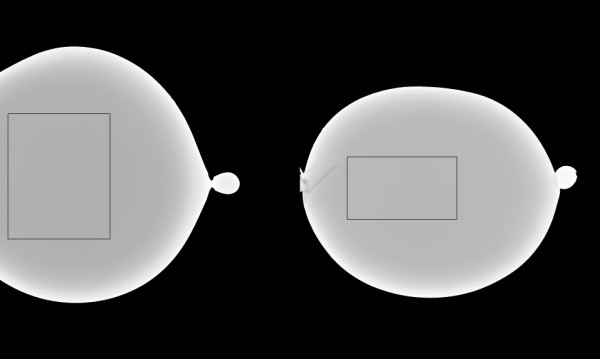
**Deformable mixture phantom examples**. The 34/66 (water/oil) mixture is shown on the left and the 31/69 mixture on the right. The calibration application was constrained to the outlined regions to avoid curvature effects. These regions are 650 ×800 pixels and 700 × 400 pixels, respectively.

**Figure 12:**
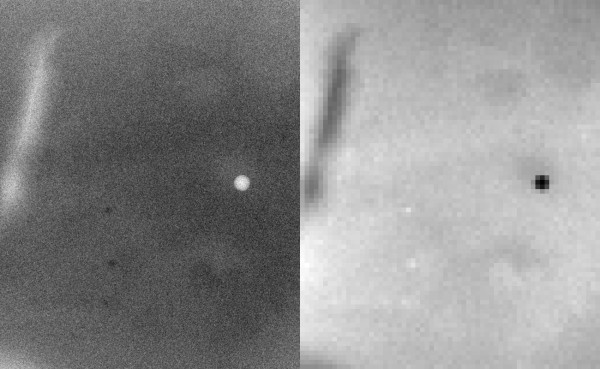
**Water/oil mixture calibration region example**. The 650 × 800 pixel region of interest outlined in Figure 11 (left image) is shown (34/66 mixture). The left figure shows the raw pixel value representation and the right figure shows the calibrated representation. For reference, percent glandular (PG) = 32-34 in the pronounced darker regions and PG = 36-37 in the lighter regions in the right figure. These slight variations are due to water-oil separation; the perceived contrast is due to the overall uniform contrast of the larger background area and does not represent large pixel value differences. The checking is due to the coarse resolution of the mapping.

We used two mixture phantoms, shown in Figure 11, to estimate the uncertainty caused by inaccurate compression thickness. The calibration was applied by dividing these larger regions into a grid of 10 × 10 pixel regions and averaging within each grid (analogous to the calibration data generation). The logarithmic response (LR) was formed by the average pixel value within each grid: LR_a_= ln(grid-average/160.0) The average corrected thickness calculated over the respective 10 × 10 grid was used for T(x, y) in Eqs. (6-8). The calibration results for the 34/66 mixture (example # 1) corresponding to Figure 11 (left) are given in Table 5, and the calibration results for the 31/69 mixture corresponding to Figure 11 (right) are given in Table 6. The percent glandular (PG) rows show the calibration with the correction. The PG_s _rows show the calibration using the system compression thickness readout, t_s _, and the PG_s+5 _rows show the calibration using t_s _+ 0.5 cm, which is a static spatial correction for comparison. The region of interest in Figure 11 (left) is shown in Figure 12 in both the raw (left) and calibrated representations (right). The thickness correction precision was estimated with the 34/66 mixture (example # 1) by performing the calibration with a small perturbation, t(x, y) +0.1 cm, added to the corrected thickness. The PG_Δ _row (Table 5 only) gives the perturbed calibration results as reference for 1 mm thickness variation. The perturbation analysis indicates the correction was within ±1 mm (average) precision. To demonstrate reproducibility, the 34/66 mixture was rotated by approximately 90 degrees (clockwise) and imaged over a range of compression forces shown in Table 7 (same format), which gave similar results.

**Table 5:** The 34/66 calibration mixture example # 1

34/66 calibration # 1					
t_s_/t (cm)	5.9/6.4	5.0/5.5	4.4/4.9	3.8/4.3	3.4/4.0
Compression force (dN)	0.0	4.0	5.0	6.0	8.0
PG (5%)	36.3 (1.1)	35.5(0.5)	35.6 (0.4)	35.6(0.4)	32.5 (0.4)
PG_s _(47%)	47.3(0.7)	49.2(0.6)	50.7(0.8)	52.5(1.0)	50.7(1.2)
PG_s+5 _(6%)	35.7(0.7)	36.4(0.6)	37.6(0.9)	37.7(0.9)	35.1 (1.1)
PG_Δ _(10%)	38.5(0.5)	37.9(0.4)	38.2(0.4)	38.4(0.4)	35.1(0.5)

The percent glandular (PG) row gives the means and standard deviations (parenthetical entries) for the calibrated pixel value distribution for the region shown in Figure 12 (right) for various sample thicknesses and compression forces by first applying the thickness correction. The PG_s _row gives the calibration results for the same region using the system thickness readout (no correction), and the PG_s+5 _row gives the calibration results by adding 0.5 cm to the system thickness readout value (simple static correction). The PG_Δ _gives the perturbed calibration by subtracting 1 mm from the corrected thickness in the calibration to assess the limits of the correction. The top row gives the system readout thickness/average corrected thickness (t_s_/t) values. The parenthetical entries in the first column are the relative $\text{difference}=\frac{\left|{\text{PG}}_{\text{m}}-{\text{PG}}_{\text{known}}\right|}{{\text{PG}}_{\text{known}}}\times 100\text{\%}$ averages taken over the respective row, where PG_m _is one of the four measured quantities and PG_known _is the known mixture composition.

**Table 6:** The 31/69 mixture calibration example.

31/69 calibration						
t_s_/t (cm)	5.5/6.0	4.5/5.0	4.0/4.5	3.5/4.0	3.0/3.6	2.5/3.2
Compression force (dN)	0.0	3.0	4.0	6.0	9.0	13.0
PG (10%)	32.6 (2.2)	33.6(1.0)	34.9 (1.0)	34.6(1.3)	34.2 (1.6)	36.3 (2.0)
PG_s _(68%)	43.6(2.2)	48.0(1.0)	50.8(1.0)	52.6(1.3)	55.3(1.5)	61.8(1.8)
PG_s+5 _(19%)	31.8(2.1)	34.5(0.9)	36.4(0.9)	37.1(1.1)	38.5 (1.4)	43.8 (1.7)

Quantities were derived from the outlined region shown in Figure 11 (right) using a similar format (see Table 5).

**Table 7:** The 34/66 calibration mixture example # 2

34/66 calibration # 2							
t_s_/t (cm)	5.3/5.8	5.1/5.6	4.5/5.0	4.1/4.6	3.7/4.3	3.4/4.0	2.9/3.5
Compression force (dN)	3.0	4.0	5.0	6.0	9.0	10.0	12.0
PG (6%)	37.6 (0.7)	36.4(1.5)	37.6 (2.1)	37.3(2.7)	35.6 (3.2)	34.2 (3.6)	33.6 (4.0)
PG_s _(55%)	49.3(2.5)	49.8(1.5)	52.6(2.2)	53.7(2.8)	54.3(3.4)	54.3(4.5)	56.4(2.5)
PG_s+5 _(14%)	37.4(2.4)	37.6(1.4)	39.3(2.1)	39.5(2.6)	39.3 (3.1)	38.5 (3.6)	39.2 (2.4)

Quantities were derived from the phantom shown in Figure 11 (left) after rotating it clockwise by 90 degrees (approximately) and re-imaging. It shows that the measurements and calibration are repeatable.

### Calibrated Representation Comparison

We compared the percent glandular (PG) and volumetric representations using Eqs. (7-8) with the polynomial correction for three system thickness readout values: 5.0 cm 4.4 cm and 3.8 cm. We retained the usage of *glandular *for comparison purposes, although water content was determined in various forms. The analysis was applied to the ROI (34/66 mixture) shown in Figure 11 (left) and in Figure 12. Using Eq. (7), the respective average glandular-volume/pixel quantities were estimated as [0.196, 0.175, 0.155] mm^3^/pixel, whereas the respective total glandular volumes were [101.9, 91.4, 80. 8] ml. These volumetric quantities changed significantly for the selected volume, whereas the PG representation was consistent (Table 5). To emphasize this finding, total fluid volumes for these examples were also estimated as [288.0, 257.9, 227.5] ml respectively. Using Eq. (8), the respective planar spatial summaries are given by: <PG> = 101.9/288.0 × 100 = 35.3, <PG> = 91.4/257.9 × 100 = 35.4, and <PG> = 80.8/227.5 ×100 = 35.5 These examples show the validity of Eq. (8) and that the PG representation is consistent with respect to thickness variations caused by applied compression force variations (Table 5).

**Figure 13:**
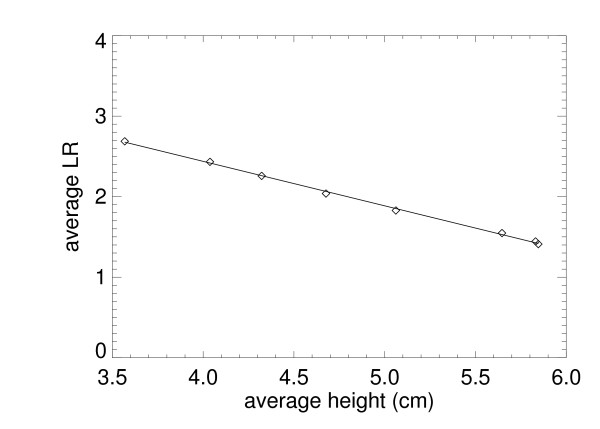
**The average logarithmic response (LR) is plotted (diamonds) for the 34/66 mixture (example # 2) taken over 25 cm^2 ^region for each height and compared with the regression fitted line (solid) **. The horizontal axis is the average corrected thickness for each region. The absolute value of the regression slope, 0.546 ± 0.01, is the effective radiation attenuation coefficient for the mixture. Letting p = 0.34, μ_e _= 0.34 × μ_w _+ 0.66 × μ_o _= 0.543, which was derived with the values from Table 4.

We used the 34/66 mixture example # 2 (because of the wider range of thickness samples) to show the relation between the PG and the Eq. (5) representation. The LR was calculated by averaging the sub-regions for each thickness (average corrected thickness over the region) in Table 7. Figure 13 shows the regression analysis findings for the 34/66 mixture (example # 2). The absolute value slope is an estimate of the average effective radiation attenuation coefficient: < μ_e _> = 0.546 ± 0.01. Using p = 0.34 as a known quantity with the effective attenuation coefficient quantities in Table 4 gives μ_e _= 0.34 × 0.708 +0.66 × 0.458 = 0.543, which is in agreement with the slope estimation. The findings for the other 34/66 sample (example # 1) gave < μ_e _> = 0.513 ± 0.01, which is also in agreement and shows that both the PG and μ_e _representations more resemble planar measures than volumetric breast density measurements.

## Discussion

The inaccurate compression thickness problem was addressed as two separate components (1) the paddle tilt due to play in the mechanical connection, which was not dependent upon the compressed sample, and (2) the paddle bulge (flex) due its elasticity and the compressed sample's resistance. Serial mechanical measurements of the paddle perimeter were approximately invariant and within ±1.0 mm precision. The H(x, y) analysis was evaluated under known conditions (precision ≤ 0.5 mm), and then used to estimate the paddle bulge. The paddle tilt and bulge assessments were used as boundary conditions for the cubic polynomial thickness correction.

We evaluated the thickness correction using methods that duplicated the rigid/non-rigid misalignment. Figure 10 shows agreement between the compressed behavior of patient mammograms and the deformable (water) phantoms. The overlap in the region between 3-8 dN illustrates the similarity. The relative calibration (average) error was reduced to 7% from 48-68% when applying the thickness correction (Tables 5, 6, 7. The thickness-corrected calibration results were in agreement with the known percent glandular (PG) quantities and within the margin of composition uncertainty. When comparing the static correction findings with those estimated with the surface correction, the latter produced calibration quantities that were closer to the known values. However, the static correction accounted for a greater portion of the overall deviation as gauged by comparing the PG and PG_s+5 _entries with the PG_s _entries (Table 5 and Table 6). This is expected because the static correction is embedded within the surface correction. The mechanical correction component was not heavily dependent upon the phantom - breast similarity. To emphasize these overall improvement gains, the average relative difference between the known and measured PG composition quantities is provided parenthetically in the first column for each of the three calibration examples (Tables 5, 6, 7). The accuracy improvements are due to the overall (average) corrected thickness precision, which was approximately within ±1 mm (Table 5). To evaluate the replication properties of both the phantom construction and the correction, the 34/66 mixture was repositioned, imaged, and calibrated, which resulted in similar findings (Table 7). The thickness correction was evaluated further by measuring the calibration parameters over a wide-area in the reference phantoms (Figure 4). The agreement between the wide-area parameters (with the correction) and those parameters measured from the strip regions (Table 4) shows the validity of the correction. In contrast, when there is thickness inaccuracy, the intercepts showed marked variation as demonstrated by comparing corrected quantities (generated from the same wide-area) with the non-corrected quantities (Table 4). We presented these findings in the PG representation because it was reproducible with respect to intra-sample thickness variations, in contrast with other volume measures.

Inaccurate compressed breast thickness is a known source of uncertainty in calibration research. Optical stereoscopic photogrammetry (OSP) methods [20,21] using stereo triangulation are also under investigation to address this problem. One variation mounted the OSP device on the mammography unit to make compressed breast measurements [21], which may not be of practical use in the clinical setting [20]. Another variation used OSP measurements of various breast models under compression to generate a thickness correction [20]. Mawdsley et al [20] found the maximum paddle height occurs at 20 mm from the chest wall (at the y-midpoint) using a system with a specific tilt-paddle. In contrast, our findings (Table 3) show the maximum occurs approximately 57 mm from the chest wall. These findings may not be directly comparable because of the differing paddle connection and operating mechanisms. Varying tilt orthogonal to the chest wall position will impact the maximum paddle height position. If the paddle front edge is fixed while increasing the tilt angle (lowering the paddle at × = x_max_), the bulge height maximum position will shift towards the chest-wall position. Moreover, the *plane *of the paddle used for our work has an upward curvature (about 1 mm crown) when resting with the maximum at approximately 73-75 mm from the chest wall slightly below the y-midpoint. The central portion of the paddle-plane also has slight but noticeable membrane characteristic when flexed with small forces. Our bulge height positions are consistent with the outer breast-paddle contact distance (~81 mm for breast and 86 mm for phantoms) when considering the plane of the paddle behaves as a deformed (bent) thin plate [27] with the load changing from a distributed load to no-load past the paddle-sample contact area. Our findings agree with Mawdsley et al [20] in that (1) the linear correction offers improved accuracy because (in this case) the offset with the system readout thickness and paddle tilt induce more variation than the paddle flex, and (2) in general there can be a significant deviation between the system readout value and the actual compressed breast thickness that requires correction. Other researchers investigated thickness inaccuracies using radio-opaque markers and magnification geometry, [19] which showed negligible deformation parallel to the chest wall and upward tilt from the breast periphery to the chest wall but to a much larger degree than indicated by Eq. (13). Our findings agree, in part, with these researchers [19] in that the deformation (near × = 0 only) parallel to the chest wall position was small; this related work did not address paddle bulge in the direction orthogonal to the chest wall position.

The calibration representation comparison showed the similarities and differences between percent glandular (PG) and related calibrated volume and height measures. The PG representation is a planar measure that is equivalent to both the normalized volumetric [11] and the normalized height [23] measurements in summary, suggesting the definitions used in the literature are not uniform. The total volume representations varied under the assumptions made here, whereas the PG measure was consistent under thickness variations for the same sample. Similar arguments apply to the total glandular height representation [7] as well.

We developed an alternative model to meet the study objectives because the phantom compositions were known. This eliminated uncertainty but its applicability relies on the similarity of the surrogate phantoms with the original model. When using mammograms to evaluate the various relationships, the compositions are unknown. Some researchers use binary labeled (breast density) mammograms [13,20] or tissue measures derived from other imaging modalities [14] in the calibration developmental work, which could introduce uncertainty. In the final validation analysis, the various calibrated measures will require a known cancer/no-cancer (CNC) endpoint to show measurement association. The developmental work could use the CNC endpoint as class separation optimization criterion for making correction adjustments, but this would preclude using the same data for independent association validation. It is less-costly to develop alternative strategies to develop and assess calibration modifications because properly designed databases that include cancer patients are time consuming and expensive to construct. The best approach is still an open ended inquiry because there is little evidence at this time showing that calibrated measurements are efficacious.

## Conclusion

The evaluation was performed with phantoms that behaved similar to that of compressed breast deformation, which is a coarse approximation. The effect of skin thickness, (if any) on the calibration accuracy was not addressed because the stretched balloon thickness was negligible compared to skin thickness, which is on the order of ~1-3 mm [28]. The overall analysis could be improved with better phantom construction methods using manufacturing techniques. The paddle bulge assessments provided an empirical (averaged) solution to loaded thin plate problem. Alternatively, the warp of the paddle plane could be estimated using numerical methods derived from plate theory [27] by (1) considering the paddle plane (thin-plate) loading of each breast separately, and (2) determining the appropriate loading geometry (eroded breast silhouette) and paddle perimeter boundary conditions. Future work includes exploring these more formal techniques of modeling the loaded paddle that could eliminate the need for the deformable breast surrogate models. Nevertheless while the deformable phantoms were less than perfect, the work showed that the thickness correction improved the calibration accuracy dramatically. Our preliminary studies were performed with homogenous phantoms, which are reasonable surrogates for developmental work but are not capable of capturing either the tissue heterogeneity present in mammograms or chest wall compression interaction. The final validation of the percent glandular measure will require a cancer/no-cancer endpoint comparison.

## Competing interests

The authors declare that they have no competing interests.

## Authors' contributions

JJH is responsible for the theoretical developments. JJH and JAT designed and constructed the surrogate system. JJH and JAT performed the experimental imaging. JJH and KC performed the data analyses and developed the computer code. JJH is the primary author. KC and JAT are secondary authors. All authors read and approved the final manuscript.
